# Valence and Motivation as Predictors of Student Time Use in Everyday Life: An Experience Sampling Study

**DOI:** 10.3389/fpsyg.2019.01430

**Published:** 2019-06-19

**Authors:** Susanne Koudela-Hamila, Axel Grund, Philip Santangelo, Ulrich W. Ebner-Priemer

**Affiliations:** ^1^Department of Applied Psychology, Karlsruhe Institute of Technology, Karlsruhe, Germany; ^2^Department of Educational Psychology, Bielefeld University, Bielefeld, Germany

**Keywords:** time use, students, electronic diary, experience sampling method, valence

## Abstract

Popular descriptions of studying frequency show remarkable discrepancies: students complain about their workload, and alumni describe freedom and pleasure. Unfortunately, empirical evidence on student time use is sparse. To investigate time use and reveal contributing psychological factors, we conducted an e-diary study. One hundred fifty-four students reported their time use and valence hourly over 7 days, both at the start of the semester and during their examination period. Motivational problems, social support and self-control were assessed once via questionnaires. Whereas the mean academic time use was in the expected range, the between-subject differences were substantial. We used multilevel modeling to separately analyze the within- and between-subject associations of valence as within factor and time use and social support, self-control, and motivation as between factors and time use. The analyses revealed the importance of affective factors on a within-subject level. Before studying, valence was already low, and it deteriorated further during studying. As expected at the between-subject level, motivational problems were related to less time studying, whereas surprisingly, self-control had no effect. The findings at the start of the semester were replicated in the examination period.

## Introduction

There are remarkable discrepancies in people’s descriptions of studying. Whereas students complain about their workload and stress, alumni describe how much they enjoyed studying and were motivated by their freedom of choice in terms of topics and courses, personal time management, and learning with other students. The latter reports support psychological and educational models and theories that link self-regulated learning to greater motivation and positive affect (Zimmerman, 2008). However, empirical evidence based on methodologically sound studies is limited, especially regarding how academic time use (i.e., time use for learning-oriented activities and course attendance) is influenced by psychological mechanisms such as valence, motivation, and self-control.

The importance of assessing academic time use has been addressed frequently in research studies (Stinebrickner and Stinebrickner, 2004; Scully and Kerr, 2014; Marshall, 2018). However, the methodological quality of these studies has often been questioned. The criticisms concern the accuracy of time use assessments, with some researchers stating that academic time use fluctuates over the course of a semester (Stinebrickner and Stinebrickner, 2004; Landrum et al., 2006; Ruiz-Gallardo et al., 2011) and that pure retrospective assessments are also problematic (Stinebrickner and Stinebrickner, 2004). In Europe, the required academic workload was specified by the Bologna reform and was set at 40 h/week. As accreditation bodies review these calculations, the effort to assess academic time use properly has further increased.

Fortunately, recent technological advances have facilitated investigating academic time use and the accompanying psychological processes prospectively in daily life. Different terms have been used to assess data in real time in daily life with these new technologies, including ambulatory assessment (Fahrenberg et al., 2007), ecological momentary assessment (Stone and Shiffman, 2002), and experience sampling method (Csikszentmihalyi and Larson, 1987). The experience sampling method (ESM) has several major advantages over measuring only once with paper–pencil questionnaires. Time use and psychological states can be captured repeatedly in everyday life in real time, thereby enabling the investigation of within-person psychological processes that may influence academic time use and avoid recall bias. Unfortunately, processes that dynamically fluctuate over time are even more prone to recall bias (Trull and Ebner-Priemer, 2013). Accordingly, academic time use, which varies considerably over an entire semester as demands change such as during the examination period (Landrum et al., 2006; Tanner et al., 2008; Ruiz-Gallardo et al., 2011), should be assessed with methods that are not prone to retrospective distortions.

In the past, paper–pencil diary studies have been used to investigate academic time use. However, accuracy of paper diaries has been questioned, as electronic timestamps are not possible. Using an electronically manipulated paper diary, Stone et al. (2002) found backfilling in 89% of all data entries. Backfilling might increase inaccuracy and might explain, in part, the wide range of absolute numbers of academic time use, ranging from 28.3 h (Nonis et al., 2006) to 50 h (Kember et al., 1996) per week in paper diary studies. Nevertheless, paper diaries might be superior to one-shot questionnaires, even though empirical evidence to support this assumption is still lacking. More recent studies use e-diaries to prevent backfilling, but to our knowledge, only three studies exist that have assessed academic time use via an electronic approach. Runyan et al. (2013) investigated the time use of undergraduate students at a Midwestern university in the US using an e-diary. The students received prompts at random times five to seven times a day between 6:00 a.m. and 11:00 p.m. Unfortunately, the assessed timeframe was time use over the last 20 min. Therefore, rather than covering the entire day of student time use, the study only examined the preceding 20 min of 5 to 7 episodes. Schulmeister and Metzger (2011) assessed time use via an online tool on which extensive backfilling was restricted electronically. They investigated daily time use in a semester over 5 months in a sample of undergraduate students. In the study, data from 18 samples from 13 different faculties at five different universities in Germany resulted in an overall academic time use of 20–27 h/week, which was far below the requested workload of 40 h/week (Metzger and Schulmeister, 2011). In a study by Marshall (2018) time use of 111 students from four different faculties at the University of New Zealand over two typical semester weeks without any deadlines was assessed. The students had to record their time use every evening on an electronic platform so that backfilling was prevented electronically. Students’ average time spent studying was 42.3 h on the first week and 40.9 h on the second week with high between-subject variability (minimum: 13.5 h and maximum: 82.0 h).

Considerable interindividual differences in academic time use have also been reported by other studies (Kember et al., 1996; Stinebrickner and Stinebrickner, 2004; Schulmeister and Metzger, 2011; Runyan et al., 2013), indicating that some students work quite a lot, whereas others do not. In addition, there is also considerable within-subject variability in academic time use, which shows that students study more during the examination period (Landrum et al., 2006; Ruiz-Gallardo et al., 2011). The differentiation of within- and between-person variance is an important step in understanding psychological processes as they unfold in daily life. The focus on between-person differences allows us to investigate whether students differ in motivation or personality traits, which could, for example, influence study time. It does not answer questions about the within-person processes that vary over time as a function of a given situation. Different situations such as the beginning of semester or the examination period, or simple daily structural patterns, could have a huge impact on behavior or mood and influence time use. Importantly, associations on different levels of analysis can differ. Referring to a famous example, the within-subject association of blood pressure and activity is positive (as walking does increase blood pressure in the moment), whereas the between-subject association is negative (as subjects with lower physical activity usually have higher blood pressure). Accordingly, it is important to separate within and between levels of analyses and to achieve representativeness, especially on the within-subject level, if this is the main level of interest. Separating between- and within-subject variance in the study design and modeling both differences statistically can be achieved using e-diaries as the assessment method and multilevel modeling as the statistical tool.

At the theoretical level, several psychological theories include assumptions about the between-subject mechanisms that influence academic time use. Models of self-regulated learning attempt to explain how students acquire knowledge and skills that encompass cognitive, motivational, and affective strategies. In particular, the concept of self-control has received considerable attention. Self-control is typically defined as “the ability to suppress prepotent responses in the service of a higher goal” (Duckworth and Seligman, 2006, p. 199). Given that self-regulated learning is often oriented toward incentives in the distant future and temptations associated with immediate pleasures (Bembenutty and Karabenick, 2004), students with a greater capacity for self-control should be able to stay on task more effectively. Moreover, findings suggest that students with greater self-control are less prone to motivational conflicts between the domains of study and leisure (Hofer et al., 2012; Grund and Fries, 2014).

Models of self-regulated learning also encompass social support (Fydrich et al., 2007), which may be helpful in terms of informative and emotional support (Aspinwall and Taylor, 1997), structure and reciprocal responsibilities (Slavin, 1996), as well as in the engagement in academic activities (Xerri et al., 2018).

The importance of motivation for successful studying in relation to well-being, adjustment to university life, perceived stress (Baker, 2004), course persistence (Vallerand and Bisonnette, 1992) and academic outcomes (Côte and Levine, 2000) has been well-documented in previous studies.

The idea that affect as another component of self-regulated learning plays an important role in the academic learning context has increased over the last years (Linnenbrink-Garcia and Pekrun, 2011; Mega et al., 2014). Nevertheless, e-diary studies involving university students are rare (Goetz et al., 2014). With regard to the *intra*personal level, recent e-diary studies among university students have shown that studying (compared to other contexts) is typically incompatible with momentary enjoyment, contentment, and positive affect (Goetz et al., 2010; Grund et al., 2015). However, whereas positive valence seems to be rare during studying, negative valence has not been addressed in previous studies. In addition, it is not yet known which mood states predict academic time use temporally.

Depending on leisure time characteristics, studies show different associations with well-being. Whereas passive leisure time (e.g., watching TV and computer-related activities done without social interaction) was negatively associated with well-being, active leisure time (e.g., social contact with friends, and physical activity) was positively associated with well-being in a traditional questionnaire study by Holder et al. (2009). Csikszentmihalyi and Hunter (2003) observed no significant association of passive leisure time, but a positive association of active leisure time with happiness in an ESM study. Leisure time in general could be associated with negative reinforcement processes, for example not having to address problems associated with learning or positive reinforcement processes such as the joy of leisure time, whereas study time serves a long-term goal and is perhaps associated with negative short-term consequences. The need for self-regulatory behavior in terms of motivation, self-control and affective states could therefore also have an impact on leisure time, which could be in the opposite direction as for academic time use.

Unfortunately, the associations between time use and psychological variables are complex, as time use is compositional and limited to 24 h a day (McGregor et al., 2018). In simple terms, not studying enhances the probability of being able to invest in leisure time. Therefore, there is a heightened probability that positive associations of psychological variables with studying coexist with negative associations of the same psychological variables with leisure time, so it is important to analyze both.

In sum, self-control, social support, motivational problems and valence, should explain interindividual differences in academic time use. Unfortunately, few studies that have assessed academic time use have separated within- and between-subject variability and used the experience sampling approach. To improve methodological quality and to generate reasonable estimates of students’ time use, we (i) assessed time use hourly using e-diaries to circumvent backfilling and retrospective distortions and (ii) assessed time use for 1 week at the start of the semester and for an additional week during the examination period to cover fluctuations over the semester.

First, we hypothesized that students’ academic time use would match the 40 h/week requirement from the Bologna Process, as accreditation boards in Europe evaluate study courses on matching this criterion and work on achieving this criterion. Second, we assumed that there would be a systematic shift in time use, with active and passive leisure time dominating the start of the semester and more time with learning-oriented activities during the examination period, as previous studies reported increased demands during the examination period (Landrum et al., 2006; Ruiz-Gallardo et al., 2011). Third, based on the finding of heterogeneity in students regarding academic time use (Kember et al., 1996; Stinebrickner and Stinebrickner, 2004; Schulmeister and Metzger, 2011; Runyan et al., 2013), we assumed that meaningful between-subject differences would exist in students’ academic time use. Summarizing the abovementioned findings on self-regulated learning, with the importance of self-control, motivation and affective factors, we hypothesized that basic psychological processes such as valence as an affective factor, self-control, social support, and motivation (or a lack thereof) could explain between- and within-subject differences in students’ time use. Similarly, we used these psychological processes to explain the variance in active and passive leisure time, as we assumed the existence of opposite effects compared to academic time use as part of compositional effects and the aforementioned opposing short- vs. long-term differences in reinforcement processes.

## Materials and Methods

### Study Design

To consider within-subject workload differences during the semester, we defined the following two measurement points: 1 week at the beginning of the semester (*start of the semester;* but not during the first 2 weeks) and 1 week during the examination period at the end of the semester (*examination period)*. The latter is in general considered the most stressful period in the German university system, since students’ grades for their courses are mainly based on the exams taken during this short period at the end of the semester. This second measurement point started for each individual 8 days before an examination and ended the day before the examination. During both weeks, students carried a smartphone (HTC Touch Diamond II©, Windows Mobile 6.5©) with them with a preinstalled e-diary. Personal-level questionnaires were administered at the start of the semester and during the examination period (see below). Data were collected at the Karlsruhe Institute of Technology (KIT) in Germany during the winter semester, which generally lasts from the middle of October to the middle of February, with an examination period of several weeks following the conclusion of the lectures in February.

Students from particular courses were asked to participate, which were selected for practical reasons (such as courses that primarily were attended by students in their first and third semesters). In groups of approximately 20, the students were informed about the study, asked to complete the first set of individual-level questionnaires, and started the e-diary. After the study week, the students returned the devices and completed another set of personal-level questionnaires, which was different from the first set. The set was divided in two to balance participant burden, because there was no reason to believe that any differences in assessment time would affect the reported data. Later in the semester, the students informed the research team about their examination dates. On that basis, appointments for the second measurement were made individually exactly 1 week before an examination. The e-diary assessment ended the evening before the examination. After the examination, the devices were returned. Again, personal-level questionnaires were completed before and after the second assessment week. The devices, e-diary questions, procedure, and timetable were the same in the examination period, except a few of the trait questionnaires that differed. To ensure compliance, the students received an individual report of their results with a personal coaching session on how to address their stressors. All students provided written informed consent. Ethical approval was not required for this study in accordance with the national and institutional guidelines.

### Subjects and Data

One hundred fifty-four students gave their informed consent. Most of the participants were male (79%) and studying industrial engineering and management (85%). The mean age of all participants was 21.1 years (*SD* = 1.5). Most of the students were in their first (25%) or third semester (51%), and 99% of the participants were German in terms of nationality. Forty-three percent of them had a part-time job. They worked a mean of 9.2 h/week (*SD* = 7.4) during the lecture period and 15.8 h/week (*SD* = 12.9) during the semester break, which includes the examination period. Due to technical problems, smartphone data from two students were lost at the start of the semester. Additionally, three smartphone datasets were lost during the examination period. Five participants dropped out before the second measurement, resulting in a final e-diary sample of 152 at the start of the semester and 146 in the examination period.

### Measurements: Momentary Data

To promote consistency throughout the paper, data from the e-diary will be labeled momentary data, whereas data from the questionnaires and the personal-level aggregated momentary data will be called personal-level data. For the statistical analysis, we refer to momentary data as within-subject data and personal-level data as between-subject data.

The e-diary emitted a signal every full hour (e.g., 9:00, 10:00, 11:00 a.m.) during the waking hours of each day during both assessment weeks. We chose such a non-random sampling scheme to improve the accuracy of the time use estimates. We assumed that it would be easier to report time use from full hour to full hour (e.g., from 9:00 to 10:00 a.m.), rather than for two random assessment points (e.g., 9:27 to 10:48 a.m.). We allowed for a 10-min maximum response delay. If the student did not answer within this time frame, the data were recorded as missing. Students put the e-diary in sleep mode before going to bed and started it again in the morning. The e-diary software MyExperience Movisens Edition (Movisens GmbH, Karlsruhe, Germany; Froehlich et al., 2007) time-stamped all responses automatically over the entire week.

#### Time Use

We used the following 10 different categories to classify time use: courses (e.g., lectures, workshops, tutorials), learning-oriented activities (e.g., reading relevant literature, thesis work, presentation preparation, literature research, explaining things to other students), other academic activities (e.g., borrowing books from the library, printing documents, organizing things at the study office), transport and idle time, household, eating and body care, job, active leisure time (e.g., sport, social contacts), passive leisure time (e.g., watching television, playing on the computer), sleeping and other activities. This allowed the students to split up the 60 (plus 10) minutes of their actual time use into the 10 categories. For example, a possible result would be 34 min of learning-oriented activities, 10 min of body care, 12 min of transport, and 7 min of idle time.

#### Valence

To assess momentary mood, we used the momentary Multidimensional Mood Questionnaire (Wilhelm and Schoebi, 2007), which has shown good sensitivity to change, good weekly reliability estimates, and good within-subject reliability estimates of *r(weekly valence)* = 0.92 and *r(within valence)* = 0.70. The within-subject predictors momentary valence and valence change and the between-subject predictor weekly valence were generated from that scale (for details, see section “Data Analysis”).

### Measurements: Personal-Level Questionnaires

#### Self-Control

We assessed self-control via the German version of the Self-Control Schedule (SCS-D; Jacobi et al., 1986), which covers the following four underlying constructs: ability to work in spite of delayed gratification, self-control of negative emotions and pain, utilization of techniques and self-verbalization regarding self-control, and belief in the controllability of one’s own life. We used the summed index of the entire scale, ranging from -93 to +93, with positive values indicating more self-control capacity. The SCS-D has good psychometric properties, with a Cronbach’s alpha of 0.82, split-half coefficient of 0.72, and test–retest reliability of 0.73 (Jacobi et al., 1986). In our study, comparable to the aforementioned results, Cronbach’s alpha was 0.80.

#### Social Support

We used the Social Support Questionnaire (FsozU; Fydrich et al., 2007) to measure perceived social support. A total score was calculated by computing the mean of the scores of the three subscales (affective support, practical support, and social integration). Ranging from one to five, higher values indicated greater social support. The FsozU has good psychometric properties, with Cronbach’s alpha values ranging from 0.81 to 0.93, a split-half coefficient ranging from 0.79 to 0.90, and satisfactory factorial and construct validity (Fydrich et al., 2007). Again, the Cronbach’s alpha of 0.93 of our study was comparable with the results mentioned above.

#### Motivational Problems

To assess a wide range of parameters that might be relevant to students’ time use, we used the student survey developed by Thiel et al. (2008). This survey was specifically designed for student time use studies and covers a wide range of relevant parameters such as motivational problems, study difficulties relating to preparation for examinations, or lack of comprehension of the study subject. For hypothesis 3, we used the following two items that addressed motivational problems in students: “I have problems motivating myself to learn” and “During learning, I often get distracted by other things.” We calculated the mean of the two items on a scale from one to eight, with higher values indicating more motivational problems (Cronbach’s alpha = 0.82).

### Missing Data and Imputation

Missing data are unavoidable in e-diaries, as completing an entry while driving a car, swimming or showering, for example, is not possible. In addition, prompting signals may not be heard in noisy environments. Additionally, e-diary software can have technical problems, or participants can be unwilling to complete the e-diary on time (e.g., during visiting lectures or while at the opera). Fortunately, standard analyses of e-diary data such as multilevel regression models automatically handle missing data. However, this is only the case if the analyses focus on within-subject effects, which is the standard case in e-diary research such as the prediction of momentary mood by stressors or time. Unfortunately, these models are unhelpful in terms of our first hypothesis as we were interested in the cumulative value (the sum instead of the mean) of academic time use over the whole week to compare it to the standard of 40 h/week. Therefore, an additional imputation procedure was necessary to obtain ratings for every waking hour to achieve an estimation of total academic time use over the week.

To take into account both within- and between-subject sources of variance, we used the following linear equation model to estimate the missing data (see Priebe et al., 2013):

Y_ij_ = Y_i_.+Y._j_–Y.., where
Y_ij_ = the estimated value of a category (Y) of a person (i) at a given timeslot (j),
Y_i_. = the person’s mean score for this category over all timeslots,
Y._j_ = the mean of a timeslot for a given category over all persons,
Y.. = the grand mean for this category over all timeslots and persons.

In some rare cases, the estimation resulted in negative values. They were negligible in number and were set to zero. In addition, if participants forgot to activate the sleep mode at night, we set the sleeping time to a maximum of 10 h and defined the remaining hours as missing data.

If the time between the previous diary entry (e.g., 9:03 a.m.) and the next entry (e.g., 10:00 a.m.) was under 60 min and caused, for example, by the delayed response of the participant in the last diary entry (e.g., 9:03 a.m.) or technical problems, we increased all the ratings proportionally to full hours. For the compliance calculation, we used a conservative approach and added these “increased” ratings to the missing data. The overall compliance (calculated as the number of missing data entries divided by completely filled data entries with a 60-min time frame) was very good at the start of the semester (80%) and at the examination period (89%). We used the imputed data only to test our first and second hypotheses but not for the multilevel model that was developed to test the third hypothesis, as relationships and not sums were the main focus.

### Data Analysis

To calculate academic time use to test hypothesis 1, we used the following two different approaches: we first calculated academic time use by summing the time used for learning-oriented activities, course attendance, and other academic activities. Academic time use in the ECTS Bologna System is defined as the estimated time that a student typically uses for learning activities, such as attending classes, projects, practical work, or independent study to reach defined learning goals (ECTS users guide, 2015). As Kember (2004) noted, the utility of a measure for course workload adding independent study is questionable as the definition of learning activities, as independent study is rather notional. In a second calculation, we included additionally half of the “transport and idle time.” We added this category, which reflects, among other things, the time required to walk from one course to another and waiting until the course starts, as these activities prevent time from being used for other activities and therefore are clearly related to studying. We used a one-sample *t*-test to compare the assessed academic time use with the 40 h/week Bologna criterion.

To compare the e-diary time use data from the start of the semester to that of the examination period, to test our second hypothesis, we used paired *t*-tests. If the assumption of normality was violated, we used the Wilcoxon test. We used visual inspection and the Kolmogorov–Smirnov test with Lilliefors-correction and a small alpha of 0.1% because of the robustness of the *t*-test against the violation of normality (e.g., Bortz, 1989). Effect size estimates were calculated using G^∗^Power©3.1.9.2 (Faul et al., 2007).

To investigate which psychological processes were related to student academic time use, our third hypothesis, we chose a more conservative approach to estimate hourly workload. We simply summed the time used with learning-oriented activities and attending courses over the previous 60 min without including the two categories of other academic activities and transport and idle time. We did this because we assumed that the time use scores of learning-oriented activities and attending courses would be more homogenous in content than the combined score of all four categories. Due to the nested data structure, we used a multilevel regression model with a three-level structure. Occasions (level 1) were nested within days (level 2), which were nested within individuals (level 3). At the beginning of the semester, the maximum data points used were 17,107 at level 1, 1,106 at level 2, and 146 at level 3. In the examination period, the maximum data points used were 17,374 at level 1, 1,052 at level 2, and 145 at level 3. One advantage of such a multilevel model is that a different quantity of data points per person can be handled and, thus, used in the analysis. To make a clear and interpretable separation of the within- and between-subject effects, we centered our variables and included (mean individual) valence as an additional personal-level variable at the between-subject level (see Nezlek, 2012) so that it described the weekly individual averages of the valence ratings assessed by the e-diary. Momentary valence was centered on the person’s mean (group-mean centering). Grand-mean centering was used for the between-subject predictors. To control for time trends, we included time as hours of the day in the model. To control for time effects that were not only linear, we also included quadratic hours as a variable in the model.

Weekend effects were controlled for using a dummy coded variable, with “weekend” coded as one and “working day” coded as zero. The control variable “hours of the day” was centered on 12 o’clock noon. We calculated six multilevel models as follows: (1) learning-oriented activities and courses at the start of the semester, (2) active leisure time at the start of the semester, (3) passive leisure time at the start of the semester, (4) learning-oriented activities and courses during the examination period, (5) active leisure time during the examination period, and (6) passive leisure time during the examination period. Even though these are different models, their outcomes are not totally independent. For example, if during a given hour, 60 min were spent on learning-oriented activities, then active and passive leisure time had to be zero. However, learning-oriented activities, courses, active and passive leisure time did not always add up to 60 min, as there were other possibilities such as housework, eating, body care, other academic activities, transport and idle time, and time invested in a part-time job. We included as level-three variables the personal-level variables of social support, self-control, and motivational problems. Full maximum likelihood estimation was used for all six multilevel models. All fixed effects from level 1 and level 2 were allowed to vary randomly.

Data management, especially the imputation procedure, was done using SAS©9.3 (SAS Institute, Inc., Cary, NC, United States). Statistical analyses for the first and second analyses were performed using SPSS©21 (SPSS, Inc., Chicago, IL, United States). For the multilevel models used to test the third hypothesis, we used HLM©7 (Raudenbush and Bryk, 2002). An alpha level of 5% (two-sided) was used for all statistical analyses.

## Results

### Time Use

**FIGURE 1:**
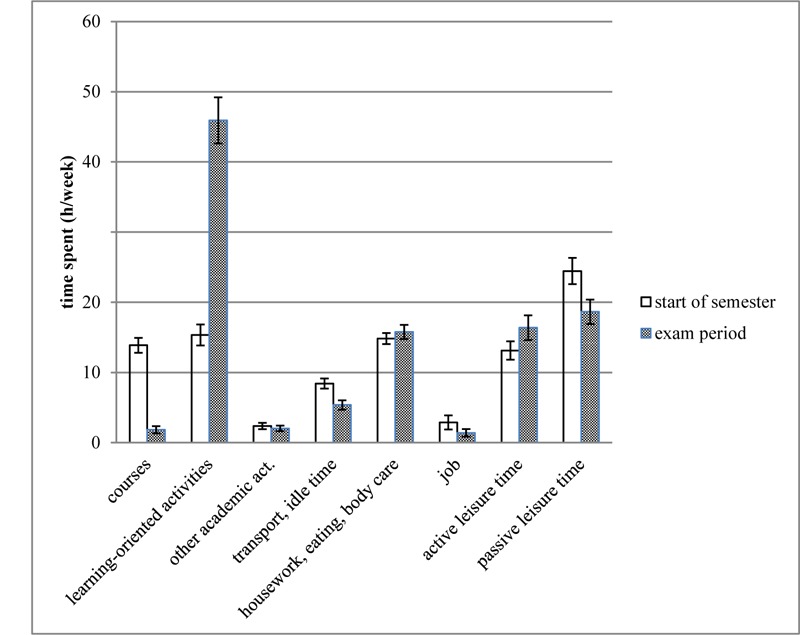
Average for each time use category from the e-diary assessment at start of semester and in the examination period (*N* = 144). The height of the bar denotes the mean and the whiskers mark the 95% confidence interval for the mean of the categories.

Figure 1 shows the time use data for both measurement points: the start of the semester and the examination period. Summing “courses,” “learning-oriented activities,” and “other academic activities” into an academic time use score for each measurement point resulted in values above and below the 40 h/week (*M*_m1_ = 31.6, *SD*_m1_ = 11.9; *M*_m2_ = 49.8, *SD*_m2_ = 20.4). Averaging both measurement points revealed a mean academic time use of 40.24 h (*SD*_m1m2_ = 13.2), which is not significantly different from the Bologna criterion, *t*(153) = 0.22, *p* = 0.824, *d =* 0.02 (hypothesis 1). However, when we added 50% of the transport and idle time to the academic time use calculation, academic time use was slightly above the criterion (*M*_m1m2transport_ = 43.7, *SD*_m1m2transport_ = 13.3, *t*(153) *=* 3.44, *p* = 0.001, *d* = 0.28). In addition, academic time use during the examination period was significantly higher, *t*(143) = -10.48, *p* < 0.001, than it was at the start of the semester, yielding a large effect (*d* = -0.87).

As predicted in our second hypothesis, Figure 1 graphically reveals a systematic shift in time use from the start of the semester to the examination period. Specifically, at the start of the semester, students spent more time engaged in courses, *t*(143) = 20.26, *p* < 0.001, *d =* 1.69, other academic activities, *z* = -2.56, *p* = 0.010, *d =* 0.22 (*Median_m1_* = 1.73; *IQR_m1_* = 2.43; *Median_m2_* = 1.22; *IQR_m2_* = 2.09), transport and idle time, *t*(143) = 7.38, *p* < 0.001, *d =* 0.61 and passive leisure activities, *t*(143) = 5.72, *p* < 0.001, *d =* 0.48, compared to the examination period. During the examination period, they spent more time engaged in learning-oriented activities, *t*(143) = -18.77, *p* < 0.001, *d =* -1.56, housework, eating, and body care, *t*(143) = -2.09, *p* = 0.038, *d =* -0.17 and active leisure activities, *t*(143) = -4.15, *p* < 0.001, *d =* -0.35, compared to the start of the semester. In the job category, no significant changes between the two measurement points were found, *z* = -0.40, *p* = 0.693, *d =* 0.25 (*Median_m1_* = 0.08; *IQR_m1_* = 3.19; *Median_m2_* = 0.11; *IQR_m2_* = 2.44). In addition, Figure 1 provides insight into the huge between-subject differences. For example, the mean time used with learning-oriented activities at measurement point two had a standard deviation of 20.4 h.

**FIGURE 2:**
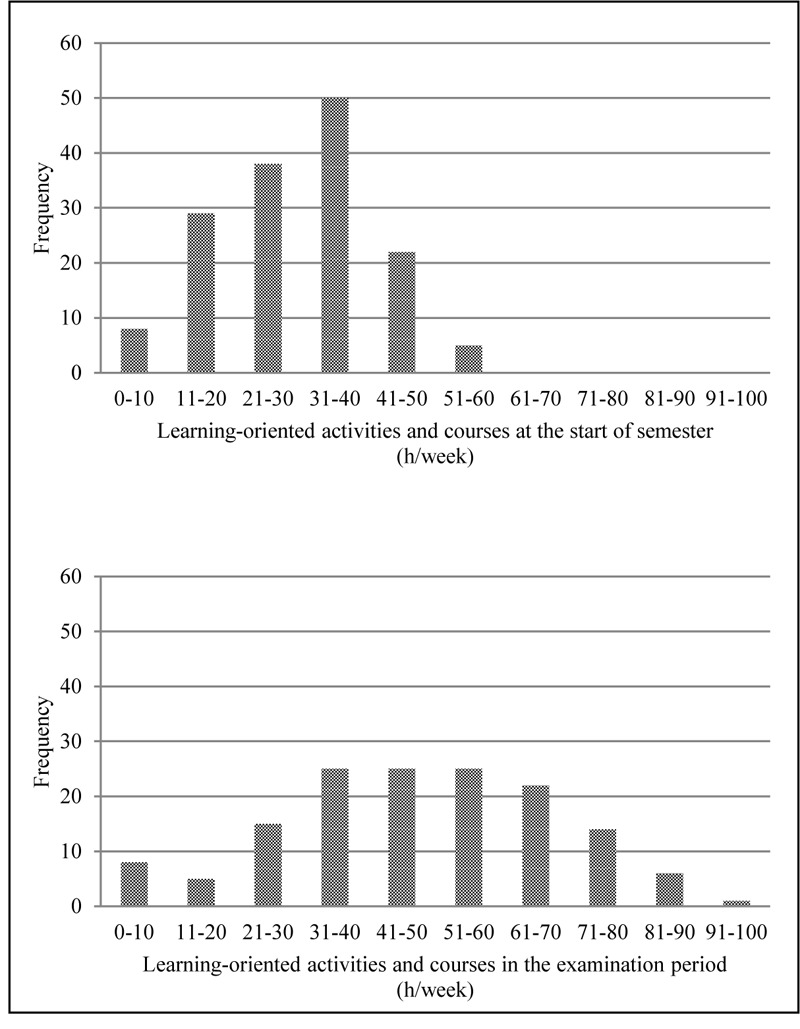
Distribution of the student’s time used for learning-oriented activities and courses (academic time use) in h/week at the start of the semester and in the examination period (*N* = 144). There are sizable differences in time spent studying per week among students. Frequency denotes number of students in each bin.

To examine between-subject differences more closely (hypothesis 3), we summed the time use scores for the learning-oriented activities and courses categories to form an academic time use score and plotted its distribution for both assessment points (see Figure 2). The range of workload for the start of the semester was already quite impressive. Specifically, students’ scores ranged from the first category “0–10 h/week” to the last category of “51–60 h/week.” The range during the examination period was nearly twice that of the start of the semester (“0–10 h/week” to “91–100 h/week”).

### Predicting Students’ Time Use

To explain between- and within-subject differences in students’ time use (hypothesis 3), we included basic psychological processes as predictor variables in six different multilevel models (see Table 1; models I–III: start of the semester; models IV–VI: examination period). We first calculated six null models to determine the two intraclass correlation coefficients (ICC2 = ICC day level; ICC3 = ICC person level) of each model at the beginning of semester [ICC2(model I) = 0.13; ICC3(model I) = 0.06; ICC2(model II) = 0.09; ICC3(model II) = 0.07; ICC2(model III) = 0.13; ICC3(model III) = 0.07] and in the examination period [ICC2(model IV) = 0.07; ICC3(model IV) = 0.11; ICC2(model V) = 0.05; ICC3(model V) = 0.07; ICC2(model VI) = 0.06; ICC3(model VI) = 0.07]. As within-subject predictors, we included valence (t_-1_), valence (t_-1_–t_0_), hours, hours^2^ and the dummy coded variable of weekend. Valence (t_-1_) describes momentary valence before the predicted time use (which is the actual time use from t_-1_–t_0_). In contrast, valence (t_-1_–t_0_) describes the change in valence during the time use of a student (for example, how mood deteriorates during learning-oriented activities). In addition, we included social support (*M* = 4.1, *SD* = 0.5), self-control (*M* = 11.3, *SD* = 19.2), motivational problems (*M* = 5.4, *SD* = 1.7), and weekly valence (*M_m1_* = 4.5, *SD_m1_* = 0.8; *M_m2_* = 4.2, *SD_m2_* = 0.9) as between-subject predictors.

**Table 1:** Results from the **m**ultilevel **a**nalysis.

	Start of semester						Exam period					
	Model I		Model II		Model III		Model IV		Model V		Model VI	
	Learning and courses^a^		Active leisure time^a^		Passive leisure time^a^		Learning and courses^a^		Active leisure time^a^		Passive leisure time^a^	
Predictors	β(SE)	*t*	β(SE)	*t*	β(SE)	*t*	β(SE)	*t*	β(SE)	*t*	β(SE)	*t*
**Between**												
Intercept	23.66 (0.66)**	35.93	3.04 (0.32)**	9.38	4.54 (0.33)**	13.68	29.62 (0.98)**	30.10	3.37 (0.44)**	7.71	5.07 (0.37)**	13.63
Social support	0.66 (0.88)	0.75	-0.83 (0.67)	-1.23	0.53 (0.72)	0.74	3.73 (1.40)**	2.66	1.49 (0.87)	-1.72	-1.10 (0.69)	-1.59
Self-control	-0.01 (0.02)	-0.35	-0.03 (0.02)	-1.79	0.01 (0.02)	0.68	0.04 (0.04)	1.07	-0.03 (0.02)	-1.42	0.00 (0.02)	0.05
Motivation^d^	-1.31 (0.28)**	-4.68	0.42 (0.16)**	2.65	0.65 (0.19)**	3.47	-1.53 (0.48)**	-3.22	0.46 (0.22)*	2.04	0.45 (0.19)*	2.38
(weekly) Valence	-2.15 (0.59)**	-3.68	0.84 (0.45)	1.87	0.72 (0.38)	1.89	-1.89 (0.84)*	-2.26	0.93 (0.56)	1.65	0.48 (0.37)	1.32
**Within**												
Valence (t_-1_)	-3.29 (0.41)**	-8.04	0.82 (0.26)**	3.21	3.35 (0.27)**	12.55	-4.00 (0.41)**	-9.81	0.79 (0.26)**	3.10	2.54 (0.25)**	10.06
Valence (t_-1_–t_0_)^b^	3.10 (0.29)**	10.89	-0.45 (0.18)*	-2.50	-2.23 (0.21)**	-10.73	2.87 (0.29)**	9.85	-0.13 (0.17)	-0.77	-1.54 (0.18)**	-8.75
**Within (control)**												
Hours	0.37 (0.05)**	7.55	0.07 (0.05)	1.49	0.23 (0.06)**	3.74	1.37 (0.11)**	12.84	0.16 (0.05)**	3.09	0.50 (0.06)**	8.39
Hours^2^	-0.16 (0.01)**	-19.58	0.09 (0.01)**	10.35	0.13 (0.01)**	13.61	-0.29 (0.02)**	-13.65	0.13 (0.01)**	12.33	0.05 (0.01)**	4.28
Weekend^c^	-8.13 (0.60)**	-13.65	3.44 (0.54)**	6.38	8.85 (0.67)**	13.17	-1.93 (0.80)*	-2.41	0.41 (0.43)	0.96	1.79 (0.50)**	3.60

*^∗^*p* < 0.05; ^∗∗^*p* < 0.001; ^*a*^learning-oriented activities and courses, active and passive leisure time: time use data from t_-*1*_–t_*0*_ in min/h; ^*b*^valence (t_-*1*_–t_*0*_): difference score of valence before minus after time use; ^*c*^weekend was dummy-coded with working day coded as zero and weekend coded as one; ^*d*^motivational problems.*

#### Time Use at the Start of the Semester (Models I–III)

We calculated three different multilevel models to predict time spent on learning-oriented activities and courses (model I), active leisure time (model II), and passive leisure time (model III) at the start of the semester (Table 1). To improve clarity, in the following section, we report the results simultaneously for all three models for each predictor, rather than for one model after another.

Momentary valence (t_-1_) did significantly predict subsequent time use (which is the actual time use from t_-1_–t_0_) in all three models (I, II, III). The association with time spent studying was negative (*p_m1_* < 0.001), whereas it was positive for active leisure time (*p_m1_* = 0.002) and passive leisure time (*p_m1_* < 0.001). This indicates that the worse the students felt, the more time they spent studying and the less time they spent engaging in active and passive leisure activities in the following hour. The negative association with studying may be interpreted as a feeling of “bad conscience.”

Changes in valence (t_-1_–t_0_) were also significantly associated with time use. More specifically, there was a significant positive effect on time spent on learning-oriented activities and in courses (*p_m1_* < 0.001), whereas there was a negative effect on time spent on active (*p_m1_* = 0.014) and passive leisure activities (*p_m1_* < 0.001). This indicates that the more the students’ valence decreased, the more time they spent studying, and the more the students’ valence increased, the more time they spent in active and passive leisure time during the hourly assessments.

Several psychological processes that were included in our multilevel model as between-subject predictors also revealed significance. Motivational problems had a significant negative effect on time spent studying (*p_m1_* < 0.001) and a significant positive effect on time spent on active (*p_m1_* = 0.009) and passive leisure activities (*p_m1_* < 0.001). The more motivational problems that students generally reported with regard to studying, the more time they spent on active and passive leisure activities and the less time they spent studying. Weekly valence, as a between-subject predictor, had a significant negative association with time spent studying (*p_m1_* < 0.001), which indicates that generally feeling bad was associated with more time spent studying. The associations between weekly valence with active and passive leisure activities were not significant. Somewhat unexpectedly, self-control and perceived social support did not have any significant effect at the start of the semester.

In addition, the weekend versus weekday differentiation did have a significant effect on all three outcomes (all *p_m1_’s* < 0.001). Students studied less, attended fewer courses, and invested more time in passive and active leisure activities on the weekends compared to weekdays. Time, modeled as hours of the day, had a positive and significant linear effect on time spent studying (*p_m1_* < 0.001) and time spent on passive leisure activities (*p_m1_* < 0.001). Non-linear effects, modeled as squared hours of the day, had a significant negative effect on time spent studying and a significant positive effect on active and passive leisure activities (all *p_m1_s* < 0.001). Taking the quadratic and the linear effect of hours of the day together, these findings indicate (when looking at an assumed waking time from 8.00 a.m. to 11.00 p.m.) that: (a) the later it was in the morning, the more time students spent studying, with a peak before midday. After that, time spent studying declined until evening; and (b) active and passive leisure time were increasing over the day with very low values until noon. To sum up, momentary valence (t_-1_) and changes in valence (t_-1_–t_0_) were significantly associated with time use. The worse students felt and the more the students’ valence decreased, the more time they spent studying, and the less time they spent in active and passive leisure time during the following hour. The more motivational problems, the more time they spent on active and passive leisure activities and the less time they spent studying. The significant between-subject predictor weekly valence revealed that generally feeling bad was associated with more time spent studying.

#### Time Use During the Examination Period (Models IV–VI)

Momentary valence (t_-1_) and changes in valence (t_-1_–t_0_) significantly predicted subsequent time use (time use from t_-1_–t_0_) in all three models (see Table 1; model IV: learning-oriented activities and courses; model V: active leisure time; model VI: passive leisure time). Similar to the findings at the start of the semester, valence had a negative effect on time spent studying (*p_m2_* < 0.001), whereas the effect on active (*p_m2_* = 0.002) and passive leisure time (*p_m2_* < 0.001) was positive. Similarly, changes in valence (t_-1_–t_0_) had a significant positive association with time spent studying (*p_m2_* < 0.001) and a significant negative association with passive leisure activities (*p_m2_* < 0.001).

Again, among all between-subject predictors, motivational problems significantly influenced time spent studying (*p_m2_* = 0.002) and active (*p_m2_* = 0.043) and passive leisure time (*p_m2_* = 0.018) in the same direction as at the start of semester. Again, weekly valence as a between-subject factor had a significant negative association with time spent studying (*p_m2_* = 0.026) but no effect on active and passive leisure time. Contrary to the start of the semester, social support had a significant positive effect on time spent studying (*p_m2_* = 0.009), indicating that the more social support that students’ perceived, the more time they spent studying hourly during the week. Once again, self-control did not show significant between-subject effects.

Controlling for time variables, the weekend effect was significant for time spent studying (*p_m2_* = 0.017) and passive leisure time (*p_m2_* < 0.001). Students studied more on weekdays and engaged in more passive leisure activities on the weekend compared to weekdays. Time, modeled as hours and squared hours of the day, had a significant association with all three outcomes (hours: time spent studying and passive leisure time: *p_m2_* < 0.001; active leisure time: *p_m2_* = 0.002; hours^2^ all *p_m2_s* < 0.001). Taking the quadratic and linear effect of hours of the day together, these findings indicate (when looking at an assumed waking time from 8.00 a.m. to 11.00 p.m.) that: (a) the later it was in the morning, the more time students spent studying, with a peak before midday. After that, time spent studying declined until evening; and (b) active and passive leisure time were increasing over the day with very low values until noon.

To sum up, all findings from the beginning of the semester were replicated except the effect of perceived social support. There was a significant effect of social support, meaning, the more social support, the more time they spent studying hourly during the week.

## Discussion

As hypothesized, on average, the academic time use of students did not differ from the Bologna criterion of 40 h/week. Even if we considered 50% of transport and idle time, the numbers were only slightly above the target value of 40 h/week. This result is in contrast to the findings of other studies that showed lower academic time use (Kozar et al., 2006; Nonis et al., 2006; Kolari et al., 2007). However, the finding is in accordance with the studies by Kember et al. (1996) and Marshall (2018), which showed that the given target value was accomplished by students. However, the generalizability of academic time use studies is generally limited by the sample. Our sample primarily consisted of male students who were studying industrial engineering and management at one university. Therefore, the results of other studies that assessed, for example, students of sociology, might differ because of the diverse fields of study. In addition, investigating the same field of study at a different university could have led to different results as well. Compared to cross-sectional research, where the major goal is to achieve a representative sample of participants, in intensive longitudinal research the primary goal is to achieve a representative sample of situations (even though achieving a representative sample of individuals is also of importance). As we used a “coverage” sampling strategy (hourly assessments during daytime querying about last hour for a whole week) and because we had good compliance, we are confident that we achieved a representative sample of situations within subjects. Even though general conclusions are limited, we want to highlight that we used a methodological approach with real-time assessment, thereby preventing backfilling and probably associated recall bias, which is especially important when the phenomena of interest fluctuate over time. Other investigations used paper–pencil diaries, so that the differing results could also be due to the different assessment strategies used, with paper–pencil diaries being prone to backfilling and recall biases. In addition, it may be speculated that the Bologna reform, with accreditation bodies’ reviewing how the 40 h are broken down for each course, may have streamlined academic time use across disciplines and universities.

The hypothesized systematic shift in the reported time use from the start of the semester to the examination period was clearly evident in our data. Across the semester, learning-oriented activities increased by nearly 20 h/week, whereas passive leisure time decreased. Surprisingly, active leisure time was higher during the examination period. Additionally, passive leisure time was still high during this period, with a mean of 18.6 h/week. Between-subject differences were huge. Almost one-quarter of the sample had an estimated academic time use for the whole semester of more than 50 h/week (using the mean of both measurement points as proxy for the whole semester). In addition, almost one-quarter of the students studied for fewer than 30 h/week. In the examination period, between-subject differences were even more pronounced.

The substantial within- and between-subject differences in academic workload allowed us to test the psychological processes that might explain these differences. Our multilevel models revealed three main findings. First, both measurement points (the start of the semester and examination period) provided highly comparable effects in association with the predictors, which increased our confidence in the findings.

Second, valence before time use was negatively associated with academic time use and positively associated with leisure time consistently across all six models. In other words, students were in a good mood before leisure time and a bad mood before learning-oriented activities. Given these findings, it seems appropriate to assume that students look forward to leisure time but approach studying uneasily. Another plausible explanation for students’ being in a bad mood before studying might be that they had studied before already. To control for that possibility statistically, we ran additional multilevel models, controlling for time use the hour before, which did not change the association between valence and studying. Moreover, valence diminished during learning-oriented activities, whereas valence improved during leisure time. Generally, these findings are in accordance with those of Goetz et al. (2010), which showed a negative association between studying and enjoyment at the momentary level. Not surprisingly, taking these findings into account, weekly valence was also negatively related to academic time use, indicating that those students who studied a lot felt worse over the course of the week. We found a smaller association of active leisure time than that of passive leisure time with valence and valence changes, indicating that more passive leisure time was associated with more positive valence and higher increases in valence than active leisure time. This finding is consistent with the idea of a differentiation between active and passive leisure time. However, the direction of the associations is contrary to the literature on passive and active leisure time and well-being (Csikszentmihalyi and Hunter, 2003; Holder et al., 2009). Differences in the assessment strategy (Holder et al., 2009) or in the analyzing procedure (aggregated within subject correlations without control variables in Csikszentmihalyi and Hunter, 2003) could explain the inconsistent findings. However, more research is needed under which circumstances and for which people passive leisure time has an advantage over active leisure time on affective states.

Third, at the between-subject level, we investigated the contribution of motivational problems, self-control, and social support. Motivational problems showed a coherent pattern across all models. Specifically, fewer motivational problems were associated with more time spent on learning-oriented activities and courses. Motivation seems to be an important psychological process that drives academic time use. Brahm et al. (2017) found a gradual decline in student motivation throughout the first academic year. This decline was less pronounced if the students did enjoy learning. They conclude that it is important to consider and try to keep enjoyment as a crucial part in student learning. Marshall (2018) found a significant correlation of *r* = 0.4 between time spent studying and motivation.

Social support was only related to academic time use during the examination period. Perhaps social support helps in the exchange of information about the study subject. It might also be that students test their level of acquired knowledge in learning groups. It might also be that social support is needed more in the form of emotional support to relieve the high level of stress in this period, which leads to more studying.

Somewhat surprisingly, self-control as a cognitive factor did not have any significant between subject associations. Given the impressive literature demonstrating the positive relationship between self-control and academic functioning (see de Ridder et al., 2012 for a recent overview), a positive effect of self-control on academic time use was expected. Restricted variance is not a plausible potential explanation, given that self-control showed meaningful variance. However, we observed a substantial correlation between self-control and motivational problems (*r =* -0.24, *p* = 0.004), indicating that students who reported fewer motivational problems also reported higher self-control. This finding is in line with the idea that motivation is a powerful predictor of academic persistence, leaving little between-person variance to be explained by more volitional concepts such as self-control (Inzlicht and Schmeichel, 2012). That is, if there is enough motivation, there is no additional need for a certain capacity of self-control. In contrast, if there is no motivation at all, then self-control alone does not appear to be of great importance to persistence in studying.

### Limitations

Even though we used a cutting-edge methodological assessment strategy that included real-time assessment with electronic devices that prevented backfilling and repeated assessment that enabled us to separate within- and between-variance components, we want to address some of the limitations of the current study. First, to ensure high compliance and low reactivity in e-diary studies, a fair balance between the number of assessment points and number of items is necessary to reduce participant burden. Given the hourly measurement points (up to 200 per individual) and the additional blood pressure and cortisol measurements (which were not reported), we had to manage participant burden by restricting the number of items and measurement weeks. Even though it would be tempting to have multiple questionnaires for each construct, we chose the student survey developed by Thiel et al. (2008) to estimate multiple relevant constructs with just one questionnaire. Similarly, we were not able to assess hourly workload during the entire 14-week semester. Fortunately, our analyses revealed meaningful associations and differences within and across constructs and, at the same time, an impressively high compliance of 80 and 89% in the two different assessment weeks.

Second, we were not able to provide empirical evidence regarding the association between workload and grades, as we could not assess the latter due to data protection and privacy issues. However, investigations of the association between academic time use and grades have generated mixed results (Dickinson and O’Connell, 2001; Plant et al., 2005; George et al., 2008). In addition, the frequently found weak associations might be attributed to biased estimates of academic time use caused by backfilling in paper diaries. Third, our students were mostly male and mainly studying industrial engineering and management, which limits the generalizability of our findings.

## Conclusion

In our sample of students in engineering sciences, the students’ average academic time use seemed to conform to the specifications and guidelines administered by the Bologna reform. Our design enabled us to reveal large between- and within-subject differences in students’ academic time use and explain these differences with psychological processes. Valence appeared to be a strong predictor of time use, highlighting the important role of affective factors in self-regulation and motivational processes of academic time use. Future research is needed to investigate the role of the consequences of negative valence on learning processes in daily life. Studies should detangle affective states that accompany the motivational, self-regulatory components of study time and the learning process. They also should investigate what emotion regulatory strategies in which context in daily life help students address mistakes in a successful manner so that positive valence can be reestablished and learning goals can be met. This could be helpful for professionals in designing appropriate interventions regarding emotion regulation in the learning process. Future studies should also enhance the understanding of within and between person variables and processes that influence academic time use further, especially regarding individual differences in within-subject relations, which may be a next step in helping to discover problematic trajectories and facilitate specific interventions.

## Ethics Statement

All students provided written informed consent. Ethical approval was not required for this study in accordance with the national and institutional guidelines.

## Author Contributions

SK-H, UE-P, and PS contributed to the design of the study. SK-H and PS performed the data collection, which was overseen by UE-P. SK-H performed and UE-P supervised data management. SK-H carried out the statistical analysis. SK-H and UE-P wrote the first draft of the manuscript. UE-P, PS, and AG wrote sections of the manuscript. All authors contributed to the manuscript revision, read and approved the submitted version.

## Conflict of Interest Statement

The authors declare that the research was conducted in the absence of any commercial or financial relationships that could be construed as a potential conflict of interest.
